# A novel wearable bioimpedance sensor for continuous monitoring of fluid balance: a study on isotonic hypovolemia in healthy adults

**DOI:** 10.1007/s10877-024-01245-z

**Published:** 2024-12-04

**Authors:** Harald Noddeland, Frida Bremnes, Anne Thorud, Katrine Rolid, Jørn Kvaerness, Ellen Andreassen Jaatun, Sigve Nyvik Aas

**Affiliations:** 1 Division of Emergencies and Critical Care, Oslo University Hospital, Oslo, Norway; 2 Mode Sensors AS, Trondheim, Norway

**Keywords:** Bioimpedance spectroscopy, Hydration, Fluid balance, Wearable sensor, Dehydration

## Abstract

Purpose: This study aimed to investigate the ability of a novel wearable bioimpedance sensor to monitor changes in fluid balance induced by furosemide. Because iso-osmotic fluid loss is expected to primarily comprise fluid from the extracellular compartment it was hypothesized that isotonic hypovolemia would increase the extracellular resistance (R_E_). Methods: 27 healthy adults (20 women, 7 men; 35 ± 10 year.) were continuously monitored by the bioimpedance sensor following administration of furosemide. Body weight, blood pressure, heart rate, sensation of thirst and selected blood parameters were tested before furosemide administration (t0), one hour (t1) and two hours (t2) after furosemide administration, and one hour after intake of a sports drink containing carbohydrate and electrolytes (t3). Urine elimination was measured throughout the intervention, and the change in extracellular fluid volume was estimated using urine elimination and established equations. Results: During hypovolemia body weight was reduced by 1.4 ± 0.2 kg (1.7 ± 0.4%). Total urine elimination during fluid loss was 1277 ± 190 mL. R_E_ increased significantly from t0 to t2 (13.6 ± 2.9%). A strong correlation was observed between the estimated change in extracellular fluid volume and the measured change in R_E_ during the isotonic fluid loss. Conclusion: This study demonstrates that the wearable bioimpedance device tested is very sensitive to furosemide-induced changes in fluid volume in healthy volunteers in a controlled environment. Additional research is needed to evaluate the ability of the device to track fluid status in a clinical setting. Trial registration: The study was registered at clinicaltrials.gov 29th of October 2021 (NCT05129358).

## Introduction

Maintaining a normal fluid balance in tissues is vital both for cellular and organ functions. Several physiological mechanisms secure homeostasis when fluid balance is challenged by gravitational stress, heat, movement, and lack of or excessive fluid intake. Many diseases and conditions are followed by disturbances in fluid balance; either loss of fluid or fluid accumulation.

Measuring tissue fluid balance is valuable both for the study of physiological functions, for monitoring of disease and for evaluation of therapy. This is particularly relevant in fields such as intensive care, chronic diseases, and sports medicine, where even minor changes in fluid balance can be critical. However, many of today’s fluid assessment methods (e.g. clinical examination, body weight, and fluid balance sheets) are either inaccurate or impractical [1, 2], which limit their utility both in primary care and hospital care. To address these limitations, there is a clear need for the development of more reliable and user-friendly methods that can seamlessly integrate into current practices.

Bioimpedance spectroscopy shows great potential as a non-invasive hydration assessment tool [3–5], and is performed by introducing a low-level, alternating current into the body or a body segment. The cell membrane separates the intracellular and extracellular space and acts like a capacitor during measurements. Its capacitive reactance is relatively high at lower frequencies, which restricts the flow of current into the intracellular space. As the frequency increases, the capacitive reactance decreases, allowing more current to pass through the intracellular compartment. Consequently, impedance measured at lower frequencies primarily reflects extracellular impedance, while impedance at higher frequencies accounts for the total impedance of both intra- and extracellular compartments [6]. Measuring impedance over a range of frequencies therefore allows for the calculation of extracellular resistance (R_E_) and total resistance (R_T_) [7], where R_E_ reflects extracellular water content and R_T_ reflects total water content [8].

Despite its usefulness, traditional bioimpedance equipment merely captures static hydration levels, lacking the practical means to monitor dynamic changes in fluid balance. Advancements in micro-electronics have enabled the development of wearable bioimpedance sensors [9], and such devices have demonstrated their ability to detect substantial changes in fluid volume during hemodialysis [10]. To our knowledge, the ability of wearable bioimpedance devices to measure smaller fluctuations in fluid volume (~ 1 L) remains largely unexplored. If wearable bioimpedance sensors are sensitive to even minor changes in fluid balance, they hold significant potential across a spectrum of patient groups where small changes can be critical.

To evaluate the efficacy of any innovative method for assessing changes in fluid status, the novel method must be compared with established reference tests. Given the absence of a universally acknowledged gold standard [11], it is necessary to employ a combination of reference tests during a controlled change in fluid volume.

The model used for inducing a controlled change in fluid volume is by the administration of the diuretic medication furosemide [12, 13]. Furosemide inhibits renal reabsorption of sodium and chloride, thereby reducing water reabsorption by the kidneys and increasing urine formation [14]. The loss of both solute and water results in minor changes in osmolality and thereby induce isotonic hypovolemia [15]. It has been demonstrated previously that whole-body bioimpedance devices are very sensitive to hypovolemia [16] but whether similar results can be obtained by a small wearable sensor measuring local bioimpedance has to our knowledge not been investigated.

Monitoring fluid balance using a local bioimpedance sensor can be divided into three key challenges. The first is determining whether the hydration status in the tissue beneath the sensor is representative of the hydration status of the whole body. The second challenge is understanding the relationship between the hydration of the tissue under the sensor and the local bioimpedance. Lastly, it is essential to evaluate whether the bioimpedance measurements can differentiate between intra- and extracellular compartments.

In the present paper we explore the response in local measurements of bioimpedance on the trunk when we introduce a whole-body reduction in extracellular fluid (i.e. moderate hypovolemia). It was hypothesized that isotonic hypovolemia would lead to an increase in the extracellular resistance (R_E_) due to a reduction in the extracellular fluid volume.

## Materials and methods

Data collection was carried out at Oslo University Hospital in the period June 2022 to November 2023, and all data collection was performed by healthcare professionals. The study was registered at clinicaltrials.gov prior to the recruitment of participants (NCT05129358).

### Study population

Healthy volunteers were recruited on the basis of the following exclusion criteria: (1) Hypersensitivity to diuretics, (2) diarrhea, (3) hypotension or orthostatic hypotension, (4) urinary retention, (5) pregnancy, (6) breastfeeding, (7) allergy to medical adhesive or hydrogel, (8) BMI < 18 or > 30, (9) any planned medical examination during the intervention period, (10) pacemaker, and 11) use of any medication with a significant effect on the body’s fluid balance. The study design, purpose and possible risks were explained to each subject before inclusion, and subjects gave their written consent to participate. Thirty-one subjects were recruited. Four subjects withdrew from the study prior to furosemide administration (three due to unrelated illness, and one due to a vasovagal syncope during the first blood sample). Characteristics for the subjects completing the intervention and included in the analyses are presented in Table 1.

**Table 1:** Subject characteristics (median and range)

Men/women	7/20
Age (yr.)	34 (23–56)
Weight (kg)	68 (53–104)
Height (cm)	168 (159–194)
Body mass index	25 (20–31)

### Investigational device

The investigational device (Re:Balans^®^, Mode Sensors AS, www.modesensors.com) is a wearable bioimpedance sensor with a form-factor of an adhesive patch.

The device consists of a printed circuit board, a coin cell battery, and hydrogel electrodes. Components are encapsulated by a protective die-cut foam and skin adhesive. The device has two outer electrodes used for current injection, and two inner electrodes used for voltage measurements. The distance between the current injecting electrodes is 120 mm, and the distance between the voltage detecting electrodes is 60 mm. The current is low (< 100µA) and below the threshold of human sensation.

A new version of the investigational device was produced after 12 subjects had been included in the study. This version was expected to be more robust against moisture and activity, and subject 13–31 used this device instead (hereafter referred to as version II). The version used by subject 1–12 is referred to as version I. The skin-contacting materials (adhesive and hydrogel electrodes) were identical for the two devices used in this study.

**Fig. 1:**
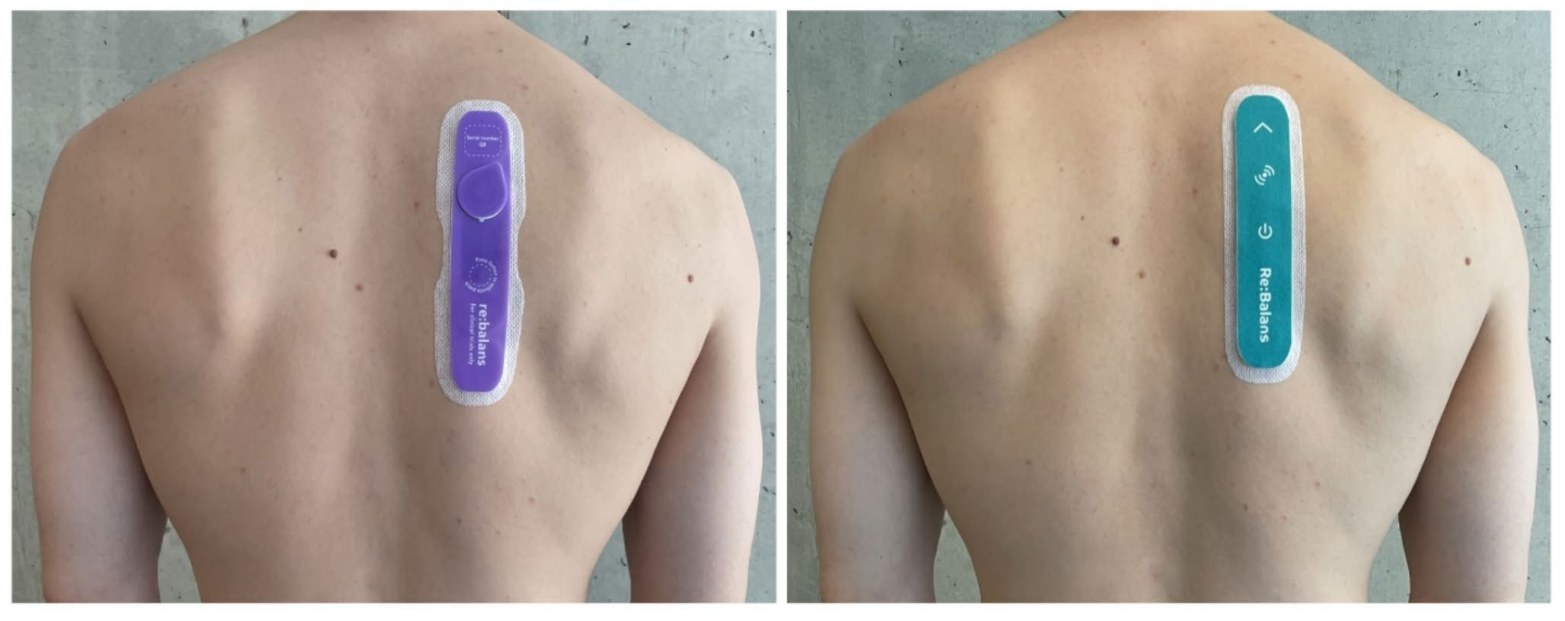
The two versions of Re:Balans^®^ used in the study. Subject 1–12 used version I (left). Subject 13–31 used version II (right)

The two versions are shown in Fig. 1.

The device utilizes a tetrapolar electrode configuration. Measurements are performed by injecting an alternating current into the tissue with the outer electrodes and measuring the voltage drop over the inner electrodes. The measured voltage and current are processed by the device’s internal circuitry and used to calculate the resulting impedance. Measurements are performed at a range of frequencies (24 for version I, 32 for version 2). The measurement accuracy is reported by the manufacturer to be within ± 2% compared to reference loads.

The absolute impedance calculated at each frequency are samples of the continuous frequency-dependent response of the underlying tissue. The investigational device utilizes an empirical model to estimate the extracellular resistance (R_E_​​) and total resistance (R_T_​) based on the absolute impedance response. The investigational device and the empirical model operate within the same frequency range as typically used for the single-cell Cole-Cole model, and the estimated R_E_ and R_T_ are closely related to the corresponding Cole-Cole parameters R0 and R∞.

In the present investigation, the device performed measurements every 30 s (subject 1–12) and 60 s (subject 13–31). The device is equipped with Bluetooth, enabling wireless transmission of bioimpedance data to a gateway or dedicated software application for real-time visualization. When out of Bluetooth range, it stores data locally on a flash memory and automatically syncs with the gateway or software application once back in range. This local storage also allows for data readout after collection is complete for further data analysis, if required, such as in clinical investigations.

The patch was placed at the upper back, between the shoulder blades (scapulas), 2–4 cm left or right of the spine. This placement is relatively close to the hydrostatic indifference point [17], which minimizes the effect of postural changes on venous pressure in the measured area. A placement on the main trunk of the body is also assumed to give an adequate representation of changes in the overall fluid balance of the body. Moreover, this is a location with less inter-individual variation in subcutaneous adipose tissue as compared to many other placements [18]. Because a tetrapolar set-up was used (four electrodes) the skin-electrode contact impedance is expected to have minimal impact on the measured impedance [19]. Based on the distance between the inner electrodes, much of the measured impedance is estimated to reflect tissue within a depth of approximately 5 cm [19, 20]. The impedance measured by the investigational device is therefore assumed to reflect primarily skin (dermis), subcutaneous adipose tissue, and muscle at this location [19, 20]. Each measurement is subject to a standardized multi-stage quality assurance (QA) process which utilizes a set of objective quality measures to determine whether measurements are to be discarded or forwarded for further processing (e.g. signal to noise ratio, spurious-free dynamic range, and battery voltage during the measurement). The development of the QA process is based on extensive analysis of a large volume of previously collected samples. The QA process was the same for both versions of the device used.

### Signal processing

During analysis of the current study, various data segments were extracted for analysis. Data segments with significant parts of the data missing due to insufficient quality had to be discarded to maintain the integrity of the analysis. For extracted data segments, a quality threshold of minimum 70% measurements passing QA was used.

After QA, the data was interpolated and filtered using a low-pass FIR filter to attenuate high-frequency changes (e.g. temporary changes caused by muscle activity). Adjustments were then made to account for any time delays introduced by the filtering process, ensuring that the timing of the signal remained reflective of the original measurements.

### Study procedures

On the first day, participants underwent initial assessments including height, weight, and blood pressure. In subject 13–31, skin thickness at the upper back was assessed by a skinfold caliper (Slim Guide, Creative Health Products, USA). A photograph of the participant’s skin was taken to establish baseline conditions before exposure to the investigational device. Next, sensors were applied to the upper back and activated. Participants were provided with diaries to record physical activities, showering, alcohol consumption, and sleep patterns. They were also instructed to promptly report any adverse events. Participants were continuously monitored by the investigational device for 8–10 days. On one of the days, subjects underwent an intervention with the diuretic medication furosemide (described below). On the final day, they returned to the lab for follow-up assessments. Diaries were collected, body weight was recorded, and the sensor patches were removed. Skin photographs were obtained, and any adverse events were registered.

### Furosemide intervention

**Fig. 2:**
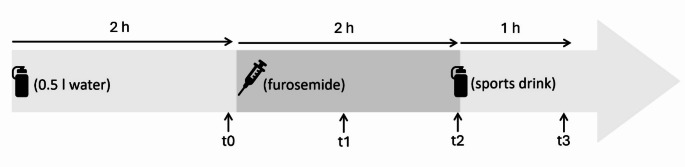
Intervention day with furosemide administration and ingestion of a sports drink

Subjects were instructed to consume 500 mL of water two hours prior to the furosemide administration, to correct any existing mild dehydration and give enough time for the fluid to equilibrate between fluid compartments. After this intake, no fluid was ingested for the next four hours. Two hours after water intake, a dose of 20 mg of furosemide was given intravenously. The aim was to induce hypovolemia corresponding to a body weight reduction of 1.5%. If weight loss was significantly less than 1.5% after one hour (e.g. <1% change in body weight), an extra dose of 10 mg furosemide was administered. Two hours after the initial dose of furosemide, subjects ingested 30 mL/kg body weight of a sports drink (Resorb^®^ Sport, Nestlé). The content per 100 mL was 2.28 g carbohydrate, 100 mg sodium, 156 mg chloride, 75 mg potassium, 48 mg calcium, and 42 mg magnesium. The maximum intake was 1500 mL, and subjects were instructed to consume the drink within 20 min. The study design is shown in Fig. 2.

Body weight, blood pressure, heart rate and sensation of thirst were assessed at t0 (before furosemide administration), t1 (one hour after furosemide administration), t2 (two hours after furosemide administration), and t3 (one hour after intake of the sports drink). Thirst was assessed using the Numeric Rating Scale (NRS; scale 1–10). Blood samples were also obtained at these four timepoints and analyzed for osmolality, hemoglobin, erythrocyte volume fraction (EVF) (Hematocrit = EVF * 100), albumin, sodium, potassium, magnesium, urea, creatinine and glucose by standard methods at the hospital laboratory (Aker Sykehus, Oslo University hospital). In addition, all urine elimination was measured between t0 and t3 using a collection container and a scale. Subjects were instructed to visit the toilet as needed, and always immediately before each measurement timepoint to ensure that the bladder was empty each time the subject was weighed. Between the measurements timepoints the participants were under observation and instructed to sit in a neutral upright position in a chair.

### Control days

The furosemide intervention timepoints were compared to corresponding timepoints on a control day. The control day was aligned with the furosemide day based on the reported wake-up times. Timepoints corresponding to t0– t3 on the intervention days are denoted c0– c3 on the control days.

The control day was selected using the following criteria: (1) The percentage of measurements passing QA from one hour before c0 to 24 h after c0 had to be above the quality threshold (70%), (2) the control day could not be the day the patch was applied, and (3) the wakeup time on the control day had to be less than 4 h apart from the wakeup time on the intervention day. After these criteria were applied, the first day fulfilling the criteria after the intervention day was used. If none of the days post intervention was within the criteria, the days before the intervention were eligible if within the criteria. One participant with a valid furosemide intervention had no valid control days. In this case the first control day with measurements within the intervention period (c0-c4) above the quality threshold was accepted for statistical analysis and plots displaying data from the intervention period.

### Calculating changes in fluid compartments

Total extracellular water (ECW) at t0 was estimated using the equation presented by Faucon and colleges [21]

$$\:ECW=\alpha\:+0.1393*w+0.0455*h+0.0125*a$$

where w denotes the body weight in kg, h the height in cm, and a the age in years. α = -2.6631 for males and − 3.3407 for females. Total blood volume was estimated using the Nadler equations [22],

$$\:{BV}_{men}=\left(0.3669*{h}^{3}\right)+\left(0.03219*w\right)+0.6041$$

$$\:{BV}_{women}=\left(0.3561*{H}^{3}\right)+\left(0.03308*w\right)+0.1833$$

where H denotes the height in meters and w denotes the weight in kg. EVF values were used to calculate plasma volume. Plasma volume was subtracted from ECW to estimate the interstitial fluid volume (ISFV) at baseline (t0). Because furosemide induces iso-osmotic fluid loss [14], the fluid loss is expected to primarily comprise extracellular fluid [15]. Total urine elimination at timepoints t1 and t2 was therefore used to estimate the ECW loss during hypovolemia.

Changes in hemoglobin and EVF during fluid loss (t0 to t2) were used to calculate changes in blood volume, plasma volume, red cell volume and mean corpuscular hemoglobin concentration (MCHC), using the equations presented by Dill and Costill [23]. These calculations allowed for the estimation of absolute and relative changes in ISFV (change in ECW not accounted for by plasma volume change).

### Statistical analyses

Baseline data, absolute and relative changes are expressed as mean ± standard deviation or median with range. Mixed effects linear models were used for comparative analysis, where timepoints were used as a factor (timepoints t0 to t3 for reference data, and timepoints t0 to t3 and c0 to c3 for bioimpedance data). Subjects were treated as random effects to account for variability across observations. The modelling and contrast testing were performed using the R packages *nlme* and *multcomp*, respectively [24, 25].

Reference data and bioimpedance data at intervention timepoints t1, t2 and t3 were compared to baseline (t0). Additionally, the rehydration period (t2 to t3) was included in the analysis. For bioimpedance data, timepoints t0 to t3 were also compared to their respective timepoints on the control day (c0 to c3) and control timepoints c1 to c3 were compared to the baseline on the control day (c0).

The ‘lme’ function from the *nlme* package was employed for mixed-effects linear modeling, and overall significance was assessed for each model using the ‘anova’ function from the *nlme* package. Upon finding significant results, contrast testing was performed using the ‘glht’ function from the *multcomp* package. The p-values were adjusted for multiple comparisons using the ‘single-step’ approach within *multcomp*. Data processing and additional analyses were performed using Python packages *NumPy*,* SciPy*,* pandas*, and *statsmodels* [26–29]. Correlations were assessed with Pearson’s correlation coefficient. A coefficient of 0.2–0.39 was considered weak, 0.4–0.69 was considered moderate, and > 0.7 was considered strong. A p-value *p* < 0.05 was considered statistically significant for all tests.

### Sample size calculations

The minimum sample size was calculated based on the primary objective and endpoint, which was the relative change in R_E_ of the upper back from t0 to t2, compared to the change in R_E_ on the reference day. A difference of 5% points was considered clinically significant. Standard deviation was assumed to be slightly higher than the difference (8), resulting in an effect size of 0.63 (mean difference / standard deviation). The sample size was then calculated using the formula

$$\:n={\left(\frac{{Z}_{1-\alpha\:/2}+\:{Z}_{1-\beta\:}}{\delta\:}\right)}^{2}$$

, where $\:{Z}_{1-\alpha\:/2}$ is the critical value corresponding to the chosen significance level, $\:{Z}_{1-\beta\:}\:$is the critical value corresponding to the desired power, and δ represents the effect size. A significance level of 0.05 and 80% power was used, resulting in a in a minimum sample size of 20 participants. However, up to 35 participants were considered for inclusion to account for any unexpected events, withdrawals or technical issues during data collection.

## Results

**Fig. 3:**
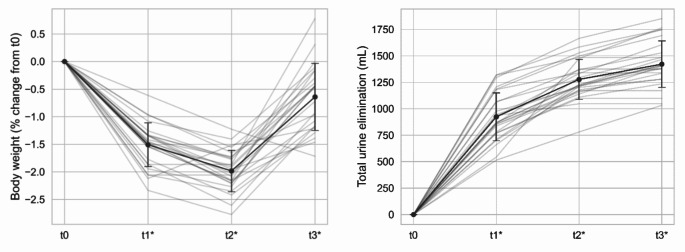
Body weight change (left) and urine elimination (right) following furosemide administration (t0 to t2) and intake of the sports drink (t2 to t3). *Significantly different from t0 (*p* < 0.05)

All 27 participants received 20 mg of furosemide at t0. Eight participants did not meet the criteria for urine elimination after one hour and received an additional dose of 10 mg at t1. During fluid loss (t0-t2) body weight was reduced by 1.4 ± 0.2 kg, corresponding to a relative loss of 1.7 ± 0.4%. The average measured total urine elimination during this period was 1277 ± 190 mL. At the same time plasma sodium concentration was unchanged, confirming that the intervention was successful in inducing mild hypovolemia (Fig. 3).

The initial body weight (t0) was correlated positively with the absolute weight loss in kg from t0-t2 (*r* = 0.43, *p* < 0.05) and the total urine elimination in mL in the same interval (*r* = 0.44, *p* < 0.05). On the other hand, there was a negative correlation between the initial body weight and the relative weight loss in % (*r*=-0.52, *p* < 0.01).

During the rehydration period (t2 to t3) the average intake of the sports drink was 1257 ± 298 mL. Not all participants were able to drink the prescribed amount, and urine elimination was 144 ± 96 mL during this phase. The average body weight after intake of the sports drink was consequently 0.45 ± 0.43 kg lower than the body weight measured at t0. Mean values of all measured reference parameters at timepoints t0-t3 are summarized in Table 2.

**Table 2:** Mean values at the four intervention time points

Timepoint	*N*	t0	t1	t2	t3
Body weight (kg)	24	73.6 ± 13.4	72.5 ± 13.3*	72.2 ± 13.3*	73.2 ± 13.3*
Total urine elimination (mL)	26	0 ± 0	924 ± 226*	1277 ± 190*	1422 ± 219*
Thirst (NRS)	26	3.5 ± 1.7	4.6 ± 1.8*	5.4 ± 1.9*	1.2 ± 1.5*
Systolic blood pressure (mmHg)	26	117 ± 12	115 ± 9	114 ± 11	120 ± 12
Diastolic blood pressure (mmHg)	26	77 ± 8	77 ± 6	76 ± 8	79 ± 7
Heart rate (BPM)	26	69 ± 9	67 ± 8	67 ± 8	62 ± 10*
Hemoglobin (g/dL)	24	13.3 ± 1.2	14.3 ± 1.3*	14.4 ± 1.3*	14.1 ± 1.2*
EVF	24	0.41 ± 0.04	0.44 ± 0.04*	0.44 ± 0.04*	0.44 ± 0.04*
Albumin (g/L)	24	44.7 ± 2.2	48.8 ± 2.4*	49.5 ± 2.6*	48.0 ± 2.7*
Sodium (mmol/L)	24	139 ± 2	139 ± 1	139 ± 2	137 ± 2*
Potassium (mmol/L)	22	4.19 ± 0.16	4.24 ± 0.37	4.26 ± 0.35	4.21 ± 0.51
Magnesium (mmol/L)	24	0.86 ± 0.07	0.89 ± 0.08*	0.9 ± 0.1*	0.86 ± 0.06
Osmolality (mmol/kg)	23	296 ± 4	298 ± 3*	299 ± 4*	295 ± 3
Urea (mmol/L)	24	5.0 ± 1.6	5.0 ± 1.6	5.0 ± 1.6	4.7 ± 1.5*
Creatinine (µmol/L)	24	73 ± 17	73 ± 17	73 ± 18	72 ± 19
Glucose (mmol/L)	24	4.7 ± 0.6	4.9 ± 0.4	4.8 ± 0.5	6.7 ± 2.4*

*Significant change from t0 (*p* < 0.05)

### Bioimpedance measurements

**Fig. 4:**
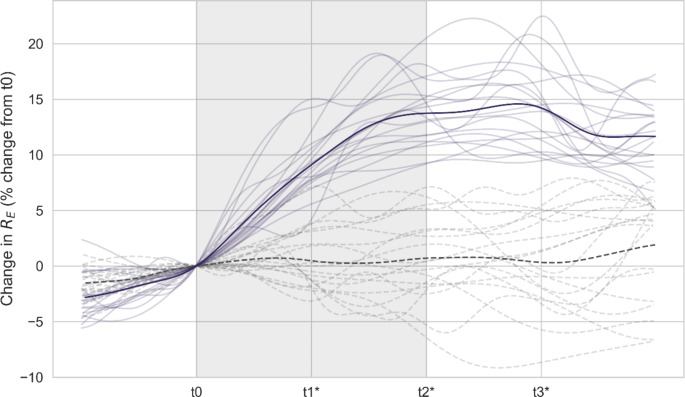
The relative change in R_E_ (%) from the start of the intervention (t0) together with control data. Furosemide was administered at t0, and the sports drink was ingested at t2. Measurements performed during the intervention are represented by solid lines. Measurements from the equivalent period on the control day are presented by dashed lines. Individual values are shown together with the average response (*N* = 19 participants shown). *Indicates significant difference between intervention timepoint and corresponding control timepoint (*p* < 0.05)

Figure 4 below displays the relative change in extracellular resistance (R_E_) at the upper back during the intervention, compared to R_E_ on a control day without any intervention. Data is plotted as % change relative to t0 and c0 for intervention and control, respectively. During the two hours of fluid loss (t0-t2), R_E_ increased significantly (13.6 ± 2.9%, *p* < 0.001). Following intake of sports drink (t2-t3), no change in relative R_E_ was observed (0.8 ± 2.7% points, *p* = 0.997). There were no significant changes in R_E_ relative to c0 during the control period c0-c3 (0.3 ± 3.7%, *p* = 0.999).

Relative changes in R_T_ displayed similar results with an increase from t0-t2 (11 ± 2.9%, *p* < 0.001), no change from t2-t3 (0.3 ± 3.1% points, *p* = 0.999), and no changes during the control period c0-c3 (-0.3 ± 3.8%, *p* = 0.999).

The primary endpoint, which was the relative change in R_E_ from t0 to t2 compared to control (c0 to c2), was 13.2% points (95% CI: 10.8 to 15.6, *p* < 0.001).

**Fig. 5:**
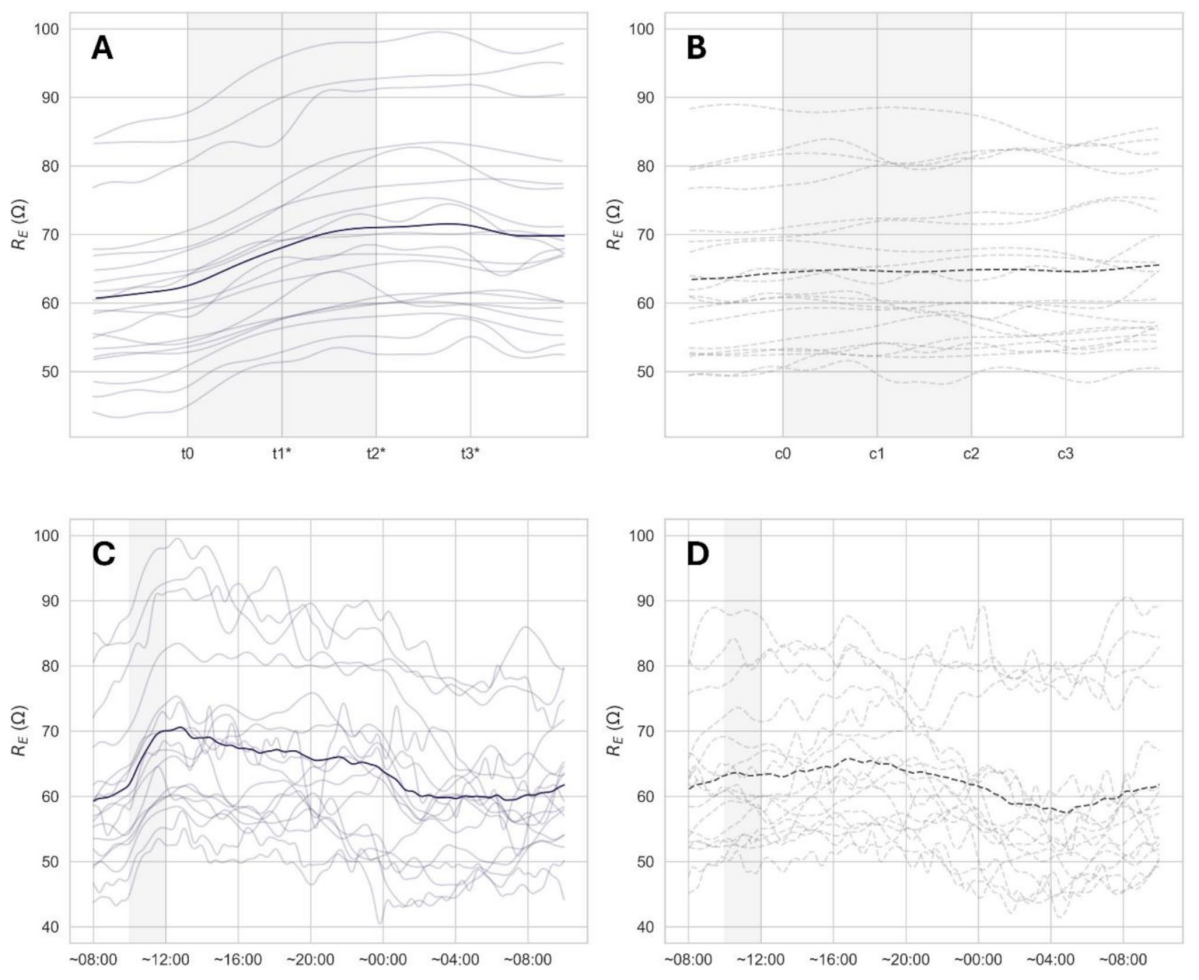
Absolute changes in R_E_ during the furosemide intervention (**A**), the corresponding time window on the control day (**B**), the 24-hour period associated with the furosemide intervention (**C**), and the 24-hour period associated with the control day (**D**). Individual values are shown together with the average response (data for *N* = 19 participants shown in panels A and B, and *N* = 17 in C and D). *Indicates significant change from t0 or c0 (*p* < 0.05)

The absolute R_E_ values are displayed in Fig. 5, showing data for the furosemide intervention (A and C), and the control day (B and D). Comparison of absolute R_E_ on the control day and the intervention day revealed no significant difference in the baseline observations at t0 compared to c0 (-2.5 ± 4.0 Ω, *p* = 0.11). This was also true for R_T_ (-0.8 ± 1.6 Ω, *p* = 0.75).

From t0-t2 R_E_ increased with 8.4 ± 2.0 Ω (*p* < 0.001), while no changes were observed following intake of sports drink (t2-t3) (0.5 ± 1.4 Ω, *p* = 0.999). R_T_ also increased from t0-t2 (4.3 ± 1.7 Ω, *p* < 0.001), followed by no change during t2-t3 (0.1 ± 0.8 Ω, *p* = 0.999). During the control period c0-c3 no significant changes were observed in either R_E_ (0.2 ± 2.5 Ω, *p* = 0.999) or R_T_ (-0.1 ± 1.6 Ω, *p* = 0.999).

Compared to their corresponding timepoints on the control day (c1-c3), absolute R_E_ was found to be higher at timepoints t1 (3.0 ± 4.1 Ω, *p* < 0.01), t2 (6.2 ± 4.9 Ω, *p* < 0.001), and t3 (6.7 ± 4.4 Ω, *p* < 0.001). The same was found for comparisons between control and intervention for R_T_ at t1 (1.9 ± 2.5 Ω, *p* < 0.01), t2 (3.6 ± 3.3 Ω, *p* < 0.001) and t3 (3.8 ± 3.3 Ω, *p* < 0.001).

Absolute and relative changes in both R_E_ and total resistance (R_T_) are tabulated in Table 3 for the timepoints during the intervention (t0-t3), and on the respective timepoints on control days (c0-c3).

**Table 3:** Absolute and relative changes in extracellular resistance (R_E_) and total resistance (R_T_) at the upper back during the intervention and control days

Timepoint	*N*	t0	t1	t2	t3
R_E_ (Ω)	19	62.5 ± 11.9	67.8 ± 12.0*	71.0 ± 13.1*	71.4 ± 13.2*
R_T_ (Ω)	19	38.4 ± 13.7	41.1 ± 14.1*	42.7 ± 15.1*	42.9 ± 15.3*
R_E_ (%)	19	0 ± 0	8.7 ± 2.8*	13.6 ± 2.9*	14.4 ± 3.9*
R_T_ (%)	19	0 ± 0	7.5 ± 2.8*	11.3 ± 2.9*	11.6 ± 3.1*
R_E_ (Ω)	19	64.6 ± 11.6	64.8 ± 11.6	64.8 ± 11.5	64.7 ± 11.6
R_T_ (Ω)	19	39.2 ± 13.2	39.2 ± 13.3	39.1 ± 13.2	39.0 ± 13.0
R_E_ (%)	19	0 ± 0	0.3 ± 2.5	0.4 ± 3.7	0.3 ± 3.7
R_T_ (%)	19	0 ± 0	0.0 ± 3.2	-0.1 ± 3.8	-0.3 ± 3.8

*Significant change from baseline (t0 or c0) (*p* < 0.05)

### Individual and inter-individual variability of R_E_ and R_T_

The variability of the measurements on the intervention day (24 h following c0) and control day (24 h following c0) was evaluated by looking at the coefficient of variation (CV) of the individual datasets and comparing them with a paired t test. There was no statistically significant difference between the CV for control and intervention data for either variable (*p* = 0.25 and *p* = 0.23 for R_E_ and R_T_, respectively).

The inter-individual variability in R_E_ and R_T_ was investigated by comparing the skin thickness with the 24-hour average of R_E_ and R_T_. Skin thickness was positively correlated with both R_E_ (*r* = 0.62, *p* < 0.05) and R_T_ (*r* = 0.87, *p* < 0.001).

### Changes in fluid compartments

Estimated changes in MCHC, blood volume, red cell volume, plasma volume, extracellular fluid volume and interstitial fluid volume on timepoints t0-t3 are presented in Table 4.

**Fig. 6:**
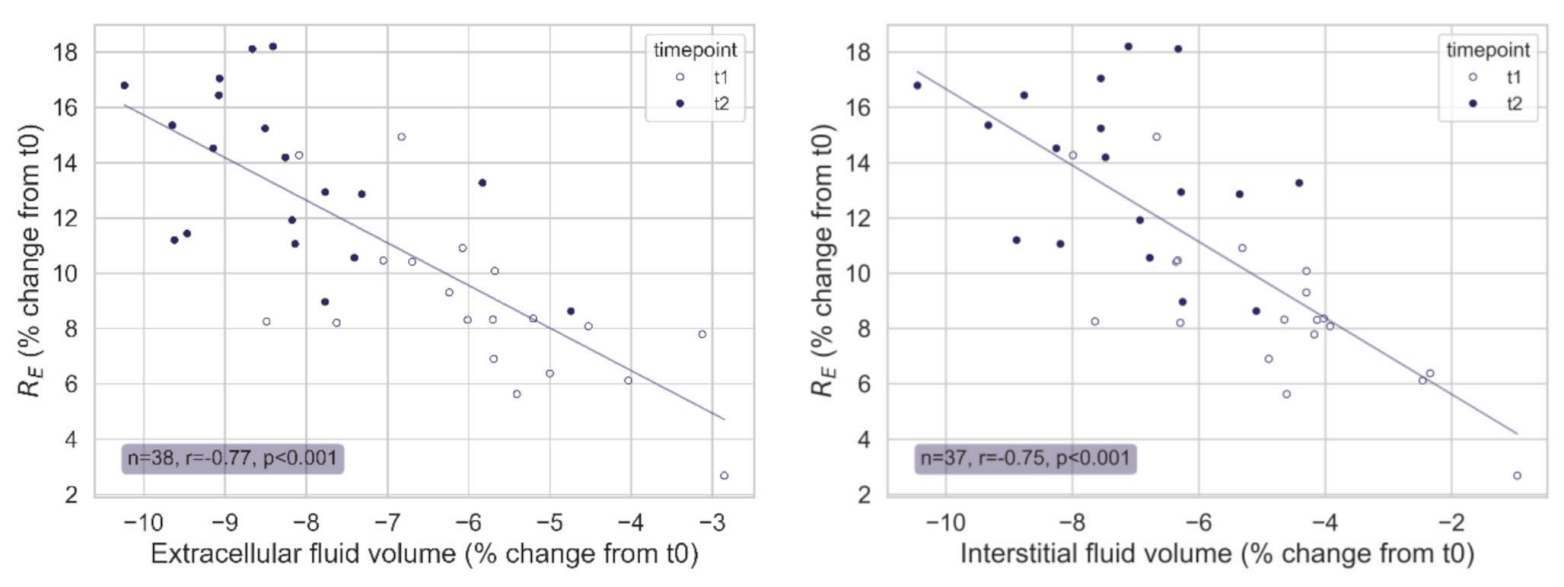
Correlation between the change in extracellular resistance (R_E_) measured by the wearable bioimpedance device and the estimated change in extracellular fluid volume (left), and interstitial fluid volume (right). Empty circles display changes from t0 to t1. Filled circles display changes from t0 to t2

A strong correlation was observed between the estimated change in extracellular fluid volume and the measured change in extracellular resistance (R) during hypovolemia (Fig. 6). The estimated change in interstitial fluid volume also displayed a strong correlation with the change in R.

**Table 4:** Absolute and relative changes in fluid compartments. Estimates are based on urine elimination, hemoglobin and EVF values (presented in table 2), using established formulas [21–23]

Timepoint	*N*	t0	t1	t2	t3
MCHC	24	32.7 ± 0.8	32.8 ± 0.8	32.6 ± 0.8	32.4 ± 0.9
Blood volume (mL)	24	4565 ± 824	4273 ± 822*	4239 ± 819*	4324 ± 846*
Blood volume (%)	24	0 ± 0	-6.6 ± 2.7*	-7.3 ± 2.7*	-5.5 ± 3.7*
Red cell volume (mL)	24	1877 ± 465	1872 ± 465	1887 ± 479	1894 ± 464
Red cell volume (%)	24	0 ± 0	-0.2 ± 2.3	0.5 ± 2.0	1.0 ± 2.9
Plasma volume (mL)	24	2688 ± 406	2401 ± 407*	2352 ± 392*	2430 ± 435*
Plasma volume (%)	24	0 ± 0	-10.9 ± 4.0*	-12.6 ± 4.0*	-9.8 ± 5.6*
Extracellular fluid volume (mL)	26	15,321 ± 2298	14,398 ± 2281*	14,044 ± 2231*	
Extracellular fluid volume (%)	26	0 ± 0	-6.1 ± 1.6*	-8.4 ± 1.3*	
Interstitial fluid volume (mL)	24	12,745 ± 1981	12,097 ± 1991*	11,798 ± 1948*	
Interstitial fluid volume (%)	24	0 ± 0	-5.2 ± 2.1*	-7.5 ± 1.72*	

*Significant change from t0 (*p* < 0.05)

The % change in R_E_ during hypovolemia (t0-t2 + t0-t1) also correlated strongly with the % change in body weight (*r* = 0.72, *p* < 0.001) and total urine elimination (*r* = 0.75, *p* < 0.001). Assuming a linear relationship between the relative change in R_E_ and urine elimination, a 1% increase in R_E_ was found to correspond to a urine elimination of 91 mL (95% CI: 0.85 to 0.98).

Changes in R_T_ correlated moderately with the % change in extracellular fluid volume (-0.63, *p* < 0.001), the % change in interstitial fluid volume (*r*=-0.62, *p* < 0.001), the % change in body weight (*r*=-0.52, *p* < 0.001), and total urine elimination in mL (*r* = 0.62, *p* < 0.001).

### Safety and user tolerance

The mean patch exposure time was 8 ± 1 days. Four cases of irritation at the patch site were reported. The causality assessment concluded that three of the reactions most likely were related to the skin adhesive. This was based on redness in the whole patch area. For one of the irritations, redness was exclusively located to the placement of the four electrodes. The most likely explanation is a reaction to the hydrogel. All irritations resolved on their own, without any need for medical attention. No serious adverse events or serious adverse device effects were reported. Some subjects reported itching the last few days of monitoring, but not to an extent where the patch had to be removed. The observations related to the safety analysis were in line with the risk assessment conducted prior to study initiation, confirming that the device is well tolerated by healthy volunteers.

## Discussion

The study shows that changes in electrical impedance measured locally on the upper back by the wearable bioimpedance sensor are closely correlated to changes in extracellular fluid when a mild isotonic hypovolemia was induced in healthy volunteers.

A similar study was conducted by the U.S. Army Research Institute of Environmental Medicine in 1999, and later retrospectively analyzed and re-published in 2016 [12, 16]. In that study, healthy volunteers were moderately dehydrated by administration of furosemide (3.5% loss in body weight). The work by Heavens et al. utilized whole body bioimpedance measurements performed at 50 kHz to investigate their resistance-reactance-score methodology. They found that changes in reactance and resistance at 50 kHz were consistent across all subjects during the intervention, both increasing significantly following furosemide-induced fluid loss. However, single “spot” measurements were not found to accurately identify dehydration due to high interpersonal variation. This agrees with the findings of the current investigation, which also demonstrated a highly consistent unidirectional response in both R_E_ and R_T_ during hypovolemia, but a significant interpersonal variation in absolute measurements. The most likely explanation for this variation between individuals is differences in the tissue composition under the patch. This is supported by the fact that both R_E_ and R_T_ correlated with skinfold thickness.

It is notable that the results in the present investigation and the study discussed above are similar even though participants in the present study are being subject to a significantly milder fluid loss, and measurements were performed locally on the back rather than on the whole body. In all individuals R_E_ rose gradually during hypovolemia, and the changes were highly correlated with reference measurements (total urine elimination, change in body weight) and estimated changes in the extracellular fluid compartment. This indicates that the sensor has a high sensitivity to changes in hydration. This also supports that although bioimpedance is measured locally on the back by the investigational device, it does reflect global changes in hydration under standardized conditions in a similar fashion as whole-body impedance measurements. This is intriguing, as the investigational device offers significant advantages in terms of usability, cost effectiveness and wearability compared to traditional whole body bioimpedance analyzers.

The potential of wearable bioimpedance devices is indeed gaining increased interest as a tool for non-invasive monitoring of fluid status [9]. Continuous monitoring is essential to capture dynamic changes in fluid status that occur during transitions between different physiological states. While wearable bioimpedance devices have been presented previously, they have typically been limited to measurements at a single frequency [10, 30], or are not practical for continuous monitoring outside the clinical setting [31, 32]. To our knowledge the investigational device presented is the first truly wearable device able to measure multifrequency bioimpedance at sub-minute resolution with a granularity high enough to differentiate between total and extracellular resistance.

Being able to estimate the extracellular resistance provides a more detailed insight into the dynamics of the fluid shifts occurring during the intervention, and the changes in extracellular resistance R_E_ were found to have a stronger association with the global changes in fluid volume during the intervention compared to the total resistance R_T_. This result is in line with the expected response to furosemide, previously reported to mainly affect the extracellular fluid volume [15, 33, 34]. The absence of significant changes in red cell volume and MCHC during the intervention also supports this, as it indicates an iso-osmotic fluid loss which mainly draws fluid from the extracellular compartment [15]. Overall, the results indicate that R_E_, as hypothesized, reflects changes in the extracellular compartment in line with the fundamental principles of multifrequency bioimpedance spectroscopy. This furthermore underscores the benefit of a device that can measure over a range of frequencies large enough to enable estimation of the extracellular resistance.

The measurements performed over the 24 h control period reveal a clear diurnal variation in R_E_, increasing gradually during daytime and decreasing back to baseline at night (Fig. 5 panel D). This corresponds to a small shift of fluid from the upper to the lower part of the body during a day in an upright position. In addition to the underlying diurnal variation, there was a notable variability in the measurements for both control and intervention days. Given the high accuracy of the bioimpedance measurements, the observed variation is likely caused by biological variations in the measured tissue. Whole body bioimpedance measurements are indeed known to be sensitive towards several factors, which is why measurements typically are taken under standardized conditions [35]. This is not always feasible, especially outside a clinical setting. Based on the observations in the present investigation, it is evident that uncompensated measurements of local bioimpedance also must be performed under standardized conditions to ensure day-to-day comparability. However, the wearability of the investigational device, high sampling frequency and granularity of the data provides an excellent foundation for timepoint selection. Nevertheless, to truly draw advantage of the vast amount of data available, the utility of the device output during non-standardized conditions should be explored in further detail.

Both R_E_ and R_T_ remained elevated after intake of the sports drink, suggesting that participants had not yet reached a normohydrated state. Other measurements taken after ingestion of the sports drink (t3) further support this, as they showed that neither body weight nor hemoglobin and EVF levels had returned to baseline values (t0). Estimated changes in blood and plasma volume also indicated that participants were still dehydrated at timepoint t3. Given that the half-life of furosemide is 0.5–2 h [14], participants were likely still experiencing the effects of furosemide during the rehydration period. This is also reflected by the continued urine elimination during this period. In addition, the sports drink was administered orally over a 20-minute period, causing delays due to both the time required for ingestion and the body’s natural absorption process of fluids. To study the effects of a rehydration intervention in greater detail, it would be beneficial to wait until the effects of furosemide have ceased before initiating rehydration.

The current investigation demonstrates that a local and wearable bioimpedance sensor is able to capture dynamic changes in global fluid balance following administration of furosemide in healthy volunteers. Given the limitations of current methods for assessing fluid status [1, 2], the successful development of a non-invasive patch that can follow changes in fluid balance would be transformative within a wide range of applications. In some use cases, an ideal device for monitoring hydration would display immediate results indicating that the tissue is normally hydrated or give a number for degree of dehydration/overhydration or hypo-/hypervolemia. Due to the individual differences in absolute measurements, more information is needed to establish normal ranges for the absolute bioimpedance measured by the device in different populations. However, in many use cases a significant deviation from a normal state may be the most important aspect of the monitoring, in which the current device shows great potential.

The measurements performed on the tissue on the back appear to adequately represent changes occurring throughout the entire body, which showcase the potential use of the sensor as a tool for remote monitoring of fluid status and potentially early detection of fluid imbalances in individuals at particular risk (e.g. elderly individuals, postoperative, chronic kidney- and heart diseases). The technology may also have significant potential within the intensive care environment where accurate management of fluid balance is crucial in the treatment of patients with acute conditions such as heart failure, kidney failure and sepsis. In this setting, the sensor can serve as an objective input and support healthcare providers in making informed decisions. However, further work is needed to validate the sensor output when used to monitor patients confined to a hospital bed. In addition to patient care, the sensor has a wide range of possible applications within research.

## Data Availability

The datasets generated in the study are available from the corresponding author upon reasonable request.
